# On pump harvesting of Left Internal Mammary Artery (LIMA) in unstable patients undergoing coronary artery bypass grafting (CABG) is a safe operative strategy: A pilot study

**DOI:** 10.12669/pjms.35.3.988

**Published:** 2019

**Authors:** Muhammad Sher-i-Murtaza, Mirza Ahmad Raza Baig

**Affiliations:** 1 *Dr. Muhammad Sher-i-Murtaza, (MBBS, FCPS). Assistant Professor of Cardiac Surgery, Ch. Pervaiz Elahi Institute of Cardiology (CPEIC), Multan, Pakistan*; 2 *Mr. Mirza Ahmad Raza Baig, (B. Sc. (Hons.) Cardiac Perfusion Technology), Clinical Perfusionist (Specialist), Cardiac Center at Hail Region, Hail, Saudi Arabia*

**Keywords:** Coronary artery bypass grafting, Left internal mammary artery, Cardiopulmonary bypass

## Abstract

**Objective:**

To evaluate the clinical safety of left internal mammary artery (LIMA) harvesting in hemodynamically unstable patients after establishing cardiopulmonary bypass (CPB) in isolated coronary artery bypass graft (CABG) surgery.

**Methods:**

The prospective observational study was conducted at Chaudhry Pervaiz Elahi Institute of Cardiology, Multan, Pakistan, from December 2016 to August 2018. All patients undergoing isolated CABG surgery in which LIMA conduit was harvested after establishing cardiopulmonary bypass because of hemodynamic instability at induction of anaesthesia or during surgery were included in the study. Preoperative, operative and postoperative characteristics of the patients were recorded. Data was analyzed using SPSS 19.

**Results:**

In Forty nine patients including 39 male and 10 female, early CPB had to be established because of hemodynamic instability and afterwards LIMA was harvested. Out of 49, 30 patients presented with CCS class III angina. 37 (75.5%) patients were scheduled on elective coronary surgery waiting list. There were 39 (79.59%) patients who weaned off bypass on mild inotropic support and 4 (8.16%) patients needed IABP support. All patients had multi-vessel coronary artery disease. Mean number of grafts were 3.428±0.577, CPB time was 110.59±25.594 and hospital stay was 5.367±1.424.

**Conclusions:**

The study showed that LIMA can be safely harvested in unstable patients after establishing extracorporeal circulation and by using this operative strategy in patients who need urgent or emergent surgical coronary revascularization LIMA can be safely used as a conduit.

## INTRODUCTION

Surgical revascularization is an effective treatment modality to treat coronary artery disease. In Coronary Artery Bypass Graft (CABG) surgery, the use of left internal mammary artery (LIMA) to bypass significant left anterior descending (LAD) artery stenosis has been proven by studies as a gold standard clinical practice. These studies have shown that LIMA graft has better long-term patency.^1^-^3^ LAD supplies major territory of left ventricle in majority of cases,^4^ hence a graft to LAD territory if remains patent for decades; benefits of CABG surgery can be prolonged resulting in better and event free long term survival. In isolated CABG, one of reason of not using LIMA is urgent or emergent surgery and hemodynamic unstable condition of the patient.^5^,^6^

We harvested LIMA in this sub group of patients after establishing cardiopulmonary bypass and used this gold standard conduit to bypass LAD stenosis. Early outcome of these patients were studied to analyze safety of this strategy.

## METHODS

This prospective observational study was conducted in department of cardiac surgery Chaudhry Pervaiz Elahi Institute of cardiology (CPEIC), Multan, Pakistan. All Patients undergoing isolated CABG surgery who were unstable or became unstable at induction of anesthesia or during or after sternotomy were included in the study.

The study was conducted from December 2016 to August 2018. Patient was considered hemodynamic unstable if he or she developed low blood pressure along with any of the following features.

- Significant STT changes in ECG monitor during induction of anaesthesia or during surgery,
- Rise in central venous pressure (CVP),
- Repeated or continuous need of inotropic support to maintain blood pressure,
- Bradycardia
- Tachycardia
- Tense PA with visible poor left ventricular (LV) contractility
- Aborted cardiac arrest

This study had approval from the ethical committee of the institution. In all our cases, median sternotomy was the approach used to access the heart for coronary bypass surgery. Heparin was administered in a dose of 400 IU/Kg. The cardiopulmonary bypass (CPB) was established with aortic and two stage right atrial cannula. Either on pump beating or cold blood cardioplegic arrest technique was used for myocardial protection in these patients. In all patients, LIMA was harvested after establishing cardiopulmonary bypass.

The inotropic support used during weaning from cardiopulmonary bypass (CPB) and in Intensive care unit (ICU) was noted It was mild when dobutamine was administrated at a rate <5 ug/kg/min, moderate when used at 5-10 ug/kg/min and high dose when dobutamine was administrated at >10 ug/kg/min. Adrenaline or nor-adrenaline dose <0.06 ug/kg/min was considered as mild support, 0.06 to 1.0 ug/kg/min as moderate and >1 ug/kg/min was considered as high inotropic support.

Maximum postoperative CK-MB levels were noted within 48 hours after surgery. A rise in CK-MB levels more than five times the reference value was considered as perioperative MI. In hospital mortality was recorded. The preoperative, operative and postoperative characteristics were summarized using mean ± standard deviation for the numeric variables and frequency for categorical variables (Table I&(II).

**Table-I:** Numeric variable of patients.

	Minimum	Maximum	Mean	Std. Deviation
Age	35.00	83.00	55.91	9.93
grafts	3.00	5.00	3.42	0.57
Clamp time*	34.00	107.00	62.73	17.86
CPB time	61.00	154.00	110.59	25.59
Ventilation time**	3.00	28.00	6.61	4.57
CKMB	18.00	169.00	57.65	26.75
ICU stay**	24.00	48.00	29.77	8.75
Hospital stay***	3.00	11.00	5.36	1.42
Parsonet	0.00	24.00	4.79	4.52
Add.Euro	0.00	8.00	1.38	1.66
Log.Euro	0.88	9.70	1.616	1.42
EF%	30.00	60.00	47.34	9.68

* minutes,

** hours,

*** days

CPB=cardiopulmonary bypass, ICU=intensive care unit, CKMB=Creatinine kinase myocardial band.

**Table-II:** Categorical variables of patients.

Name of variable	Number of patients	percentage
Total number of patients	49	100%
***Gender***		
Male	39	79.6
Female	10	20.4
***Angina Class***		
Class II	5	10.2
Class III	30	61.2
Class IV	14	28.6
***LV Systolic Functional Grade***		
Grade I	24	49.0
Grade II	13	26.5
Grade III	12	24.5
***Priority of surgery***		
Elective	37	75.5
Urgent	10	20.4
Emergent	2	4.1
***IABP***		
Not used	45	91.8
Per-op	3	6.1
Pre-op	1	2.0
***Inotropes***		
Mild	39	79.6
Moderate	8	16.3
High	2	4.1

## RESULTS

In forty-nine (49) patients including 39 males and 10 females (3.9:1), LIMA was harvested after establishing cardiopulmonary bypass. Majority of patients who became unstable during surgery was presented with angina CCS class III (61.2%) and 75.5% was on elective priority list. Out of 49, 39 (76.9%) patients weaned off from bypass on mild inotropic support. In 45 (91.8%) patients there was no need of IABP (intra aortic balloon pump). Only four patients needed IABP per-operatively. Forty five patients underwent conventional CABG and four patients had on pump beating heart CABG surgery. All patients had multi-vessels coronary artery disease and mean graft applied was 3.42±0.57. Mean clamp time was 62.73±17.86 minutes and mean bypass time was 110.59±25.594. Mean hospital stay of studied patients was 5.36±1.424 days. One patient expired on 6^th^ postoperative day due to respiratory failure. Operative mortality was 2.04%. One patient developed perioperative Myocardial infarction.

## DISCUSSION

Studies have revealed that in patients requiring urgent or emergency revascularization the rate for LIMA usage was 61.5% compared with 92.5% in the non-emergent group.^7^This is a fairly common reason for non-usage of LIMA. The lower rate of LIMA usage in this group of patients is likely to be due to hemodynamic instability or a greater risk of bleeding (as these patients are often on antithrombotic and antiplatelet therapy).^5^,^6^

In routine surgical practice, LIMA is harvested before establishing extracorporeal circulation or cardiopulmonary bypass. Patients with severe multi-vessel CAD and tight Left main stem stenosis can become unstable during induction of anesthesia or during surgery.^8^Routine clinical practice in these patients is to establish urgent cardiopulmonary bypass, and only vein conduit is used to bypass all significant coronary stenotic lesions which is easy and rapid to harvest in such scenario. Hence these patients are deprived of LIMA graft and its long term benefits. Literature review has revealed that in such emergent situation, experienced surgeons can harvest the LIMA in a relatively short time^6^ but this not always safe to harvest LIMA in presence of hemodynamic instability and under shear stress. It may jeopardize safety of patient because of inadequate organ perfusion due to hemodynamic instability and can result in damage to LIMA because of hustle. Many studies have shown that early establishment of extracorporeal circulation is affective and useful even in patients with emergency cardiac arrest.^9^,^10^

After establishing CPB there is significant reduction of myocardial O_2_ consumption of empty beating heart. As reported by Allen BS and colleagues that the O_2_ requirement of beating empty heart is reduced more than 50% as compared to the beating filled heart.^11^ Taking the concept from these studies, when we established cardiopulmonary bypass in unstable patients, the body perfusion is supported and maintained by a pump and not by heart.

We believe that LIMA can be safely harvested after establishing cardiopulmonary bypass in hemodynamically unstable patients without compromising systemic organ perfusion and safety of the patients. After establishing cardiopulmonary bypass, heart becomes empty; there is a decrease in LVEDV, wall tension, work load and oxygen demand. Force of contractility is reduced by avoiding the use of catecholamine to maintain systemic perfusion in ischemic heart while maintaining adequate coronary perfusion on pump.^12^

### Limitation of this study

It is small sample size study. So there is a need to conduct larger multi-center studies to determine the adequacy of on-pump LIMA harvesting in hemodynamically unstable patients.

## CONCLUSION

Based on the results of this study we believe that in urgent or emergent condition left internal mammary artery can be safely harvested and hemodynamic instability is no longer a relative contraindication for LIMA harvesting.

### Authors’ Contribution

**MSM** conceived, designed, did data collection and manuscript writing.

**MARB** did statistical analysis & editing of manuscript.

**MSM & MARB** did review and final approval of manuscript.
